# Host diversity and landscape structure drive genospecies-specific Lyme disease risk across Eurasia

**DOI:** 10.1016/j.onehlt.2026.101593

**Published:** 2026-09-22

**Authors:** Yifan Sun, Yinsheng Zhang, Jinchen Wang, Ruying Fang, Yiyang Guo, Sen Li

**Affiliations:** a School of Environmental Science and Engineering, Huazhong University of Science and Technology, Wuhan 430074, PR China; b Institute of Artificial Intelligence, Huazhong University of Science and Technology, Wuhan 430074, Hubei, PR China

**Keywords:** *Borrelia burgdorferi* sensu lato, Dilution effect, Host diversity, Landscape heterogeneity, Fragmentation, Tick-borne disease

## Abstract

Lyme disease poses an escalating global health challenge partly driven by climate change and land-use transformation, while mechanistic understanding of transmission remains limited. We analyzed prevalence records from 43 Eurasian countries to map pathogen biogeography and applied theory-driven structural equation modeling (SEM) to evaluate hypothesized direct, indirect, and cascading pathways linking climate, land cover, landscape structure, and host biodiversity to pathogen prevalence. Genospecies exhibited distinct biogeographic patterns shaped by vector-host specificity, with *Ixodes ricinus* dominating transmission in Europe and *I. persulcatus* in Asia. *Borrelia afzelii* prevalence was associated with climate, forest fragmentation, and landscape heterogeneity, and exhibited a negative association with rodent species richness at the continental scale. *Borrelia garinii* showed no host diversity effects but responded directly to temperature and landscape structure, reflecting reliance on highly mobile avian hosts. These findings reveal fundamental transmission heterogeneity among genospecies arising from divergent host-reservoir ecologies, with critical implications for disease control. Effective surveillance must integrate genospecies-specific ecology with landscape management to align biodiversity conservation, climate adaptation, and public health.

## Highlights

• Continental-scale *Borrelia* analysis across 43 Eurasian countries (2528 records).
• *B. afzelii declines with rodent richness; B. garinii shows no host diversity effect.*
• Fragmentation creates opposing pathways: amplification vs. diversity promotion.
• *B. garinii* driven by temperature and landscape diversity.
• Genospecies-stratified SEM reveals transmission heterogeneity.

## Graphical abstract

**Figure:**
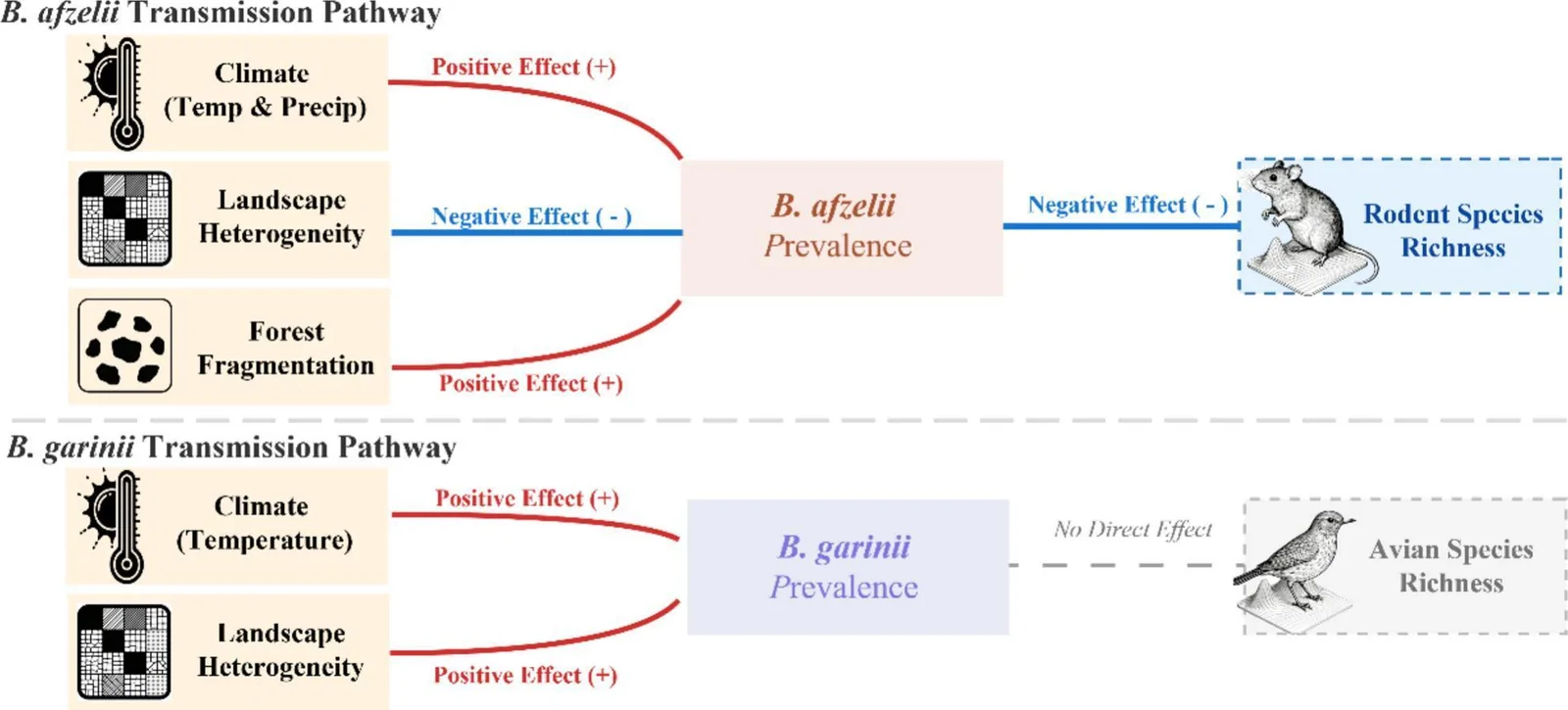

## 1 Introduction

Lyme disease, caused by *Borrelia burgdorferi* sensu lato (*B. burgdorferi* s.l.), is the most prevalent tick-borne disease in the Northern Hemisphere and a growing concern for global public health [1], [2]. Each year, approximately 476,000 people are diagnosed with and treated for Lyme disease in the United States, and an estimated 232,000 cases occur annually in Western Europe [3]. New foci of transmission are also emerging across Asia [4], [5]. These patterns have been linked to wide-ranging anthropogenic environmental changes that can alter interactions among ticks, hosts and humans, facilitating the expansion of vector populations and circulation of pathogens [6], [7], [8], [9]. Understanding how these environmental dynamics govern pathogen transmission is essential for anticipating future disease risks and developing proactive public health interventions.

*Ixodes* ticks, the primary vectors of *B. burgdorferi* s.l., undergo three life stages (larva, nymph, adult) through obligate blood-feeding on vertebrate hosts. This obligate host-feeding behavior enables pathogen acquisition from infected hosts and subsequent transmission across life stages, making tick-host interactions central to disease ecology. Climate warming can influence seasonal tick activity and range boundaries while restructuring host abundance and community composition [10], [11]. Land-use change, including forest fragmentation and agricultural intensification, reshape landscape patterns and modulate tick-host contact and pathogen transmission dynamics [9], [12], [13]. However, the magnitude and direction of these environmental effects may vary across pathogen genospecies. *B. burgdorferi* s.l. comprises a species complex of over 20 genospecies, among which *B. afzelii* and *B. garinii* represent the predominant human pathogens in Eurasia [14], [15]. *B. afzelii* depends mainly on rodent hosts and is often linked with dermatological disease, whereas *B. garinii* utilizes avian hosts and is associated with neurological infection [15], [16]. Their distinct host associations indicate different ecological pathways of transmission. Clarifying these genospecies-specific environmental drivers is critical to understanding transmission dynamics and developing precise and effective interventions for Lyme disease.

Despite extensive investigation, key uncertainties persist. Research on *c* ecology remains fragmented and seldom integrates findings across continents [17], [18]. Although some studies have characterized the prevalence distribution of specific genospecies in Europe [19], [20], [21] and localized regions of Asia [18], we still lack integrated understanding of how vector turnover, dispersal corridors, and climatic gradients shape genospecies ranges. Existing research on the drivers of pathogen prevalence is largely confined to local-to-regional scales, focusing on abiotic drivers (such as climate change) [19], [22], and only a few studies have incorporated critical biotic factors, such as host community data obtained from field surveys [23], [24]. The results from such models are difficult to generalize across broader geographical extents. Mechanisms remain unresolved, with current research seldom distinguishing direct effects from indirect and cascading effects of different environmental factors. This mechanistic uncertainty impedes understanding of transmission pathways and limits robust inference on transmission ecology. Host biodiversity adds another layer of complexity. In North America, research provides strong empirical support for the dilution effect of biodiversity in *Borrelia burgdorferi* sensu stricto (*B. burgdorferi* s.s.) transmission, where abundant non-competent hosts limit infection among competent reservoirs such as the white-footed mouse (*Peromyscus leucopus*) [25], [26]. However, the dilution effect hypothesis remains controversial in Eurasia. Complex host-vector-pathogen systems challenge generalization and have impeded integration of biodiversity into disease surveillance frameworks [27], [28]. This gap impedes both our understanding of how biodiversity moderates disease dynamics and the development of ecosystem-based prevention strategies.

The ecology of *B. burgdorferi* s.l. involves interconnected processes through which climate and land cover may influence pathogen prevalence both directly and indirectly via landscape structure and reservoir-host communities. Structural equation modeling (SEM) has been widely applied to complex ecological systems because it can represent an a priori conceptual hypothesis as a network of interrelated pathways and simultaneously evaluate direct, indirect, and cascading associations [29], [30], [31].

This study maps the distribution of *B. burgdorferi* s.l. and major pathogenic genospecies across Eurasia, evaluating associations between different genospecies and dominant tick genera. Structural equation modeling (SEM) is used to analyze genospecies-specific transmission pathways, quantifying direct, indirect, and cascading effects of climate, land cover, landscape, and host diversity on pathogen prevalence in ticks. Host diversity is assessed according to genospecies-specific ecology, distinguishing small mammal communities relevant to *B. afzelii* and avian communities associated with *B. garinii*. Through this integrative analysis, the present study provides a theory-informed foundation for developing genospecies-specific risk prediction and informing integrated management strategies linking biodiversity conservation, climate resilience, and planetary health protection.

## 2 Methods

We used a comprehensive dataset of *B. burgdorferi* s.l. prevalence that we compiled and previously published [32]. This dataset integrates 2528 prevalence records from 522 publications covering 43 countries across Eurasia for the period 2000–2023. Our prior publication provides a detailed account of the systematic literature search, data extraction, and quality control methods [32].

Potential drivers of pathogen prevalence were categorized into four domains: climate, land cover, landscape, and host diversity. All variables were resampled to a uniform 5 arc-minute spatial grid (approximately 10 × 10 km at the equator) to ensure spatial consistency. Prevalence for each record was calculated as the number of *Borrelia*-positive ticks divided by the total number of ticks examined within the corresponding sampling unit. Climate variables included temperature of the wettest quarter and precipitation of the warmest quarter, obtained from the WorldClim database (version 2.1) [33]. Land cover variables comprised forest cover percentage and built-up area percentage, derived from the EarthEnv database [34]. Landscape metrics included forest fragmentation index and landscape diversity. The forest fragmentation index was calculated as the equal-weighted average of three key landscape metrics (edge density, patch density, and mean patch area) [35]. Landscape diversity was quantified using the Shannon diversity index, calculated from MODIS Land Cover data via moving window analysis [36]. This metric integrates both the richness of patch types and the evenness of their spatial distribution within each window, capturing the heterogeneity of landscape composition.

Host diversity variables were taxonomically stratified according to species' roles in tick-borne pathogen transmission. Three host categories were defined: (1) avian hosts, including ground-foraging and migratory species from the orders Passeriformes, Galliformes, and Charadriiformes; (2) small mammal hosts, comprising rodents (order Rodentia); and (3) medium-to-large mammal hosts, including species from the orders Artiodactyla, Carnivora, Lagomorpha, and Perissodactyla. Geographic range data for all species within these taxonomic groups were obtained from the International Union for Conservation of Nature (IUCN) Red List database. Species richness for each host category was calculated through spatial overlay of species range polygons at the 5 arc-minute resolution. Detailed information on data sources and predictor variables is provided in Table S1.

We employed SEM to assess the direct, indirect, and cascading effects of climate, land cover, landscape diversity, and host diversity on pathogen prevalence in ticks. SEM was implemented as a system of linear structural equations, with all variables standardized prior to analysis. Model parameters were estimated using maximum likelihood (ML). Although ML-based SEM assumes multivariate normality, assessment of normality indicated substantial departures from this assumption in the present dataset. To address this violation, the Bollen-Stine bootstrap procedure was used to assess overall model fit under non-normality [37]. Bias-corrected 95% bootstrap confidence intervals were estimated from 2000 resamples (random seed = 1) for the direct and indirect pathways. Tick species identity was not incorporated as a random effect, as the analytical platform (AMOS 24) does not support mixed-effects SEM structures.

Based on ecological theory and published evidence, we specified an a priori path model (full model) linking climate, land cover, landscape structure, host richness, and pathogen prevalence (Fig. 1, Fig. S1 and the accompanying table). The full model incorporated all hypothesized pathways. Following initial model fitting, non-significant paths (*P* > 0.05) were sequentially removed to derive parsimonious final models. In addition to *B. burgdorferi* s.l., we constructed separate structural equation models for *B. afzelii* and *B. garinii.* Model goodness-of-fit was evaluated using the chi-square-to-degrees-of-freedom ratio (χ^2^/df) and the root mean square error of approximation (RMSEA). For *B. burgdorferi* s.l., χ^2^/df = 1.678 and RMSEA = 0.027; for *B. afzelii*, χ^2^/df = 1.934 and RMSEA = 0.039; for *B. garinii*, χ^2^/df = 1.383 and RMSEA = 0.026, indicating acceptable model fit.

**Fig. 1:**
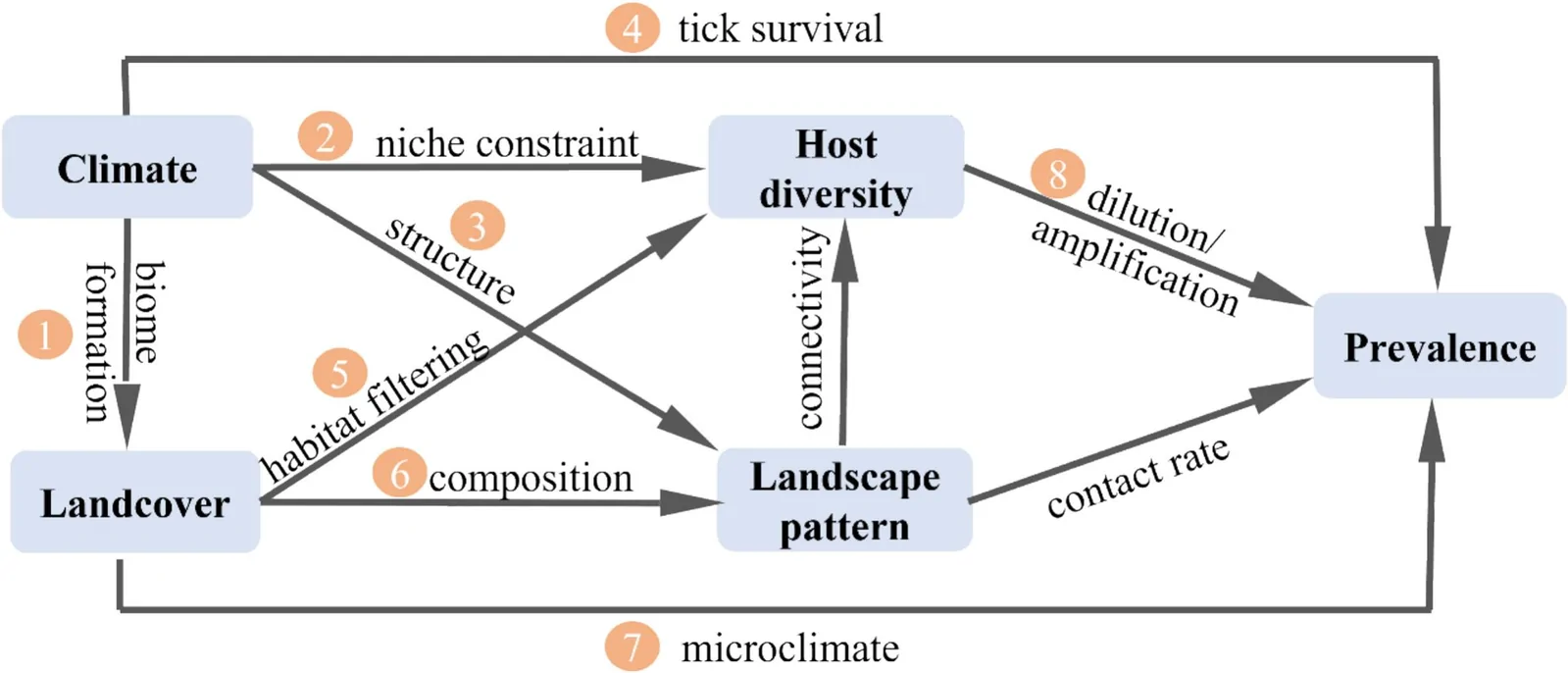
The a priori conceptual model of hypothesized relationships between drivers and *Borreli*a prevalence.

All spatial data processing, extraction, and mapping were conducted in ArcGIS Pro 3.1.4 (Esri, Redlands, CA, USA). All SEM analyses were conducted using IBM SPSS Amos 24 (IBM Corp., Armonk, NY, USA).

## 3 Results

### 3.1 Continental surveillance and prevalence patterns

Our dataset comprised 2528 prevalence records from 43 countries across Eurasia (Fig. S2). Records were reported at five administrative levels, from national to fourth-level administrative divisions. Western and Central European countries predominantly reported fine-scale data, while Asian countries showed coarser spatial resolution, reflecting regional disparities in tick surveillance and research capacity. Sampling intensity was highly uneven, with the highest record density in Central and Western Europe (Germany, Poland, Czech Republic and United Kingdom, etc.). In Asia, China contributed most records. However, vast regions of Central and Northern Asia remained undersampled. Mean national prevalence of *B. burgdorferi* s.l. varied substantially, with the highest rates observed in Malaysia and Moldova.

### 3.2 Biogeography of key vectors and genospecies

Vector-pathogen associations differed strongly among tick genera (Fig. 2). *Ixodes* spp. served as the primary vector, accounting for the majority of positive records and demonstrating widespread distribution across Europe and Asia. *Haemaphysalis* spp. showed more restricted infection patterns, concentrated in East Asia, particularly China. *Dermacentor*, *Rhipicephalus*, and *Hyalomma* spp. had very few positive records and very low prevalence, suggesting a minor role in *Borrelia* transmission in this region.

**Fig. 2:**
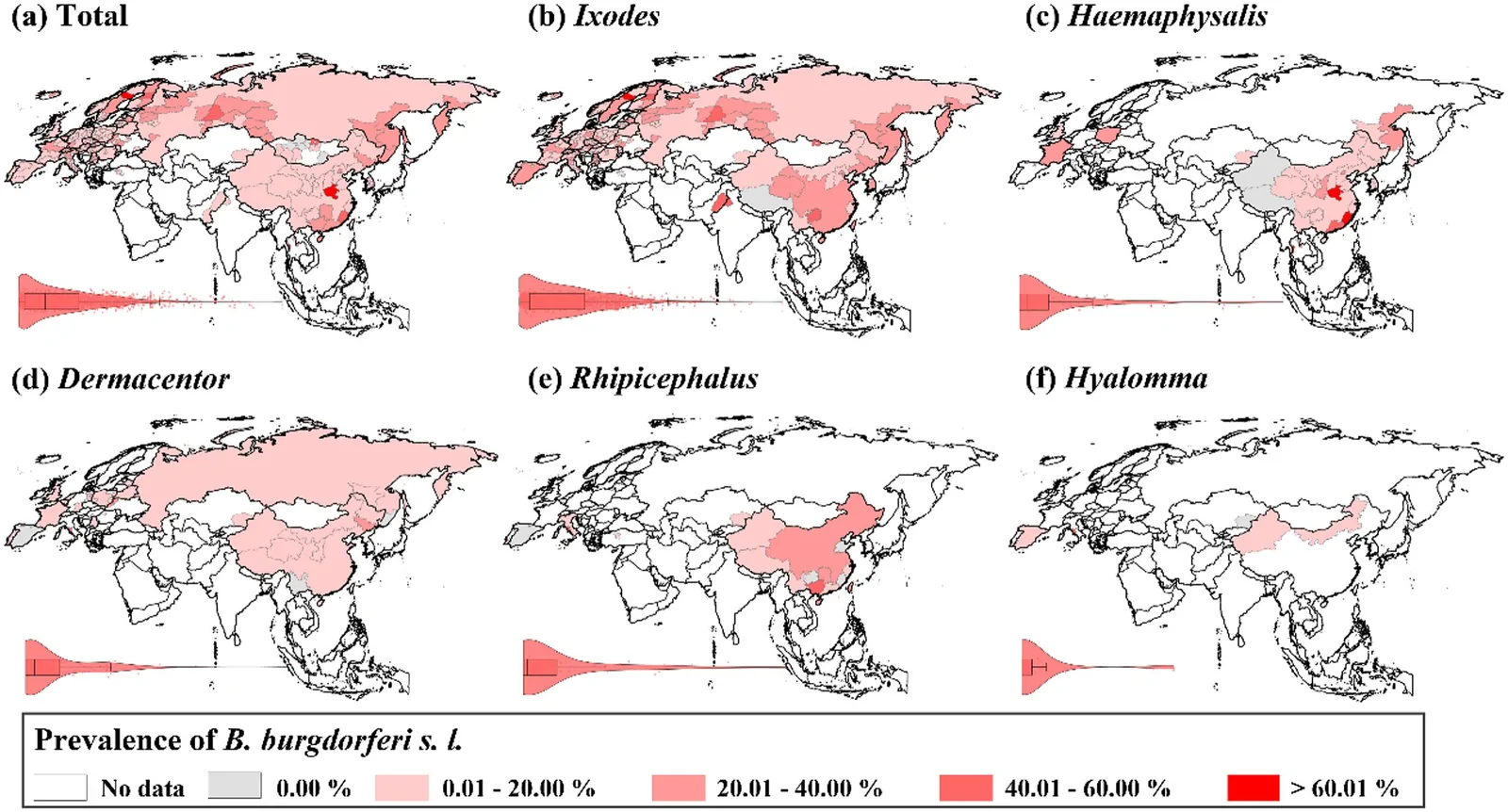
Geographic (map) and statistical (raincloud plot) distribution of *B. burgdorferi* s.l. prevalence in different tick genera across Eurasia. (a) Overall prevalence distribution in ticks. (b-f) Genus-specific prevalence distribution in *Ixodes*, *Haemaphysalis*, *Dermacentor*, *Rhipicephalus* and *Hyalomma*, respectively.

Genospecies showed distinct biogeographic patterns (Fig. 3). *B. afzelii* was dominant in Central and Western Europe, with sporadic occurrence in East Asia. *B. garinii* exhibited the broadest geographic range, extending from Western Europe through Russia to East Asia, with higher prevalence in northeastern China. *B. burgdorferi* s.s. was largely confined to Western Europe, while *B. valaisiana*, *B. lusitaniae*, and *B. spielmanii* showed restricted distributions.

**Fig. 3:**
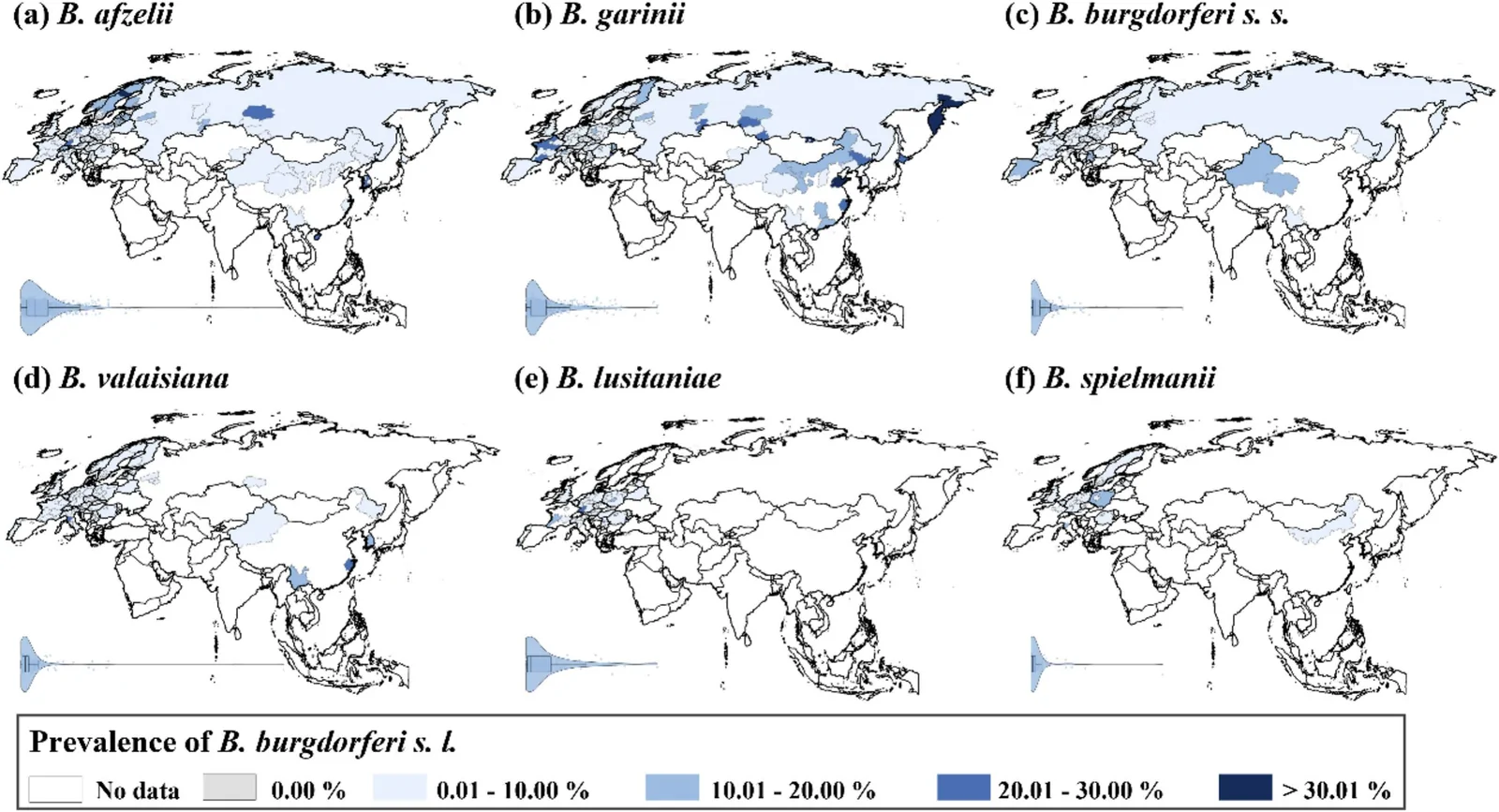
Geographic (map) and statistical (raincloud plot) distribution of prevalence in different genospecies across Eurasia. (a-f) *B. afzelii*, *B. garinii*, *B. burgdorferi* s.s., *B. valaisiana*, *B. lusitaniae* and *B. spielmanii*, respectively.

Among the 18 identified genospecies, *B. afzelii* and *B. garinii* were the most prevalent and widespread (Fig. 4). *B. afzelii* showed its highest prevalence (2–5%) in *I. ricinus*, while *B. garinii* achieved 10–20% prevalence in *I. pavlovskyi*. Co-infections occurred across *Ixodes* species, with *I. ricinus* harboring the greatest genospecies diversity. *Haemaphysalis* spp. showed limited but consistent *B. garinii* infection in East Asia. *Dermacentor*, *Rhipicephalus*, and *Hyalomma* species had prevalence below 2%. Prevalence also varied significantly across tick life stages and behavioral states, with detailed patterns presented in the appendix (Fig. S3).

**Fig. 4:**
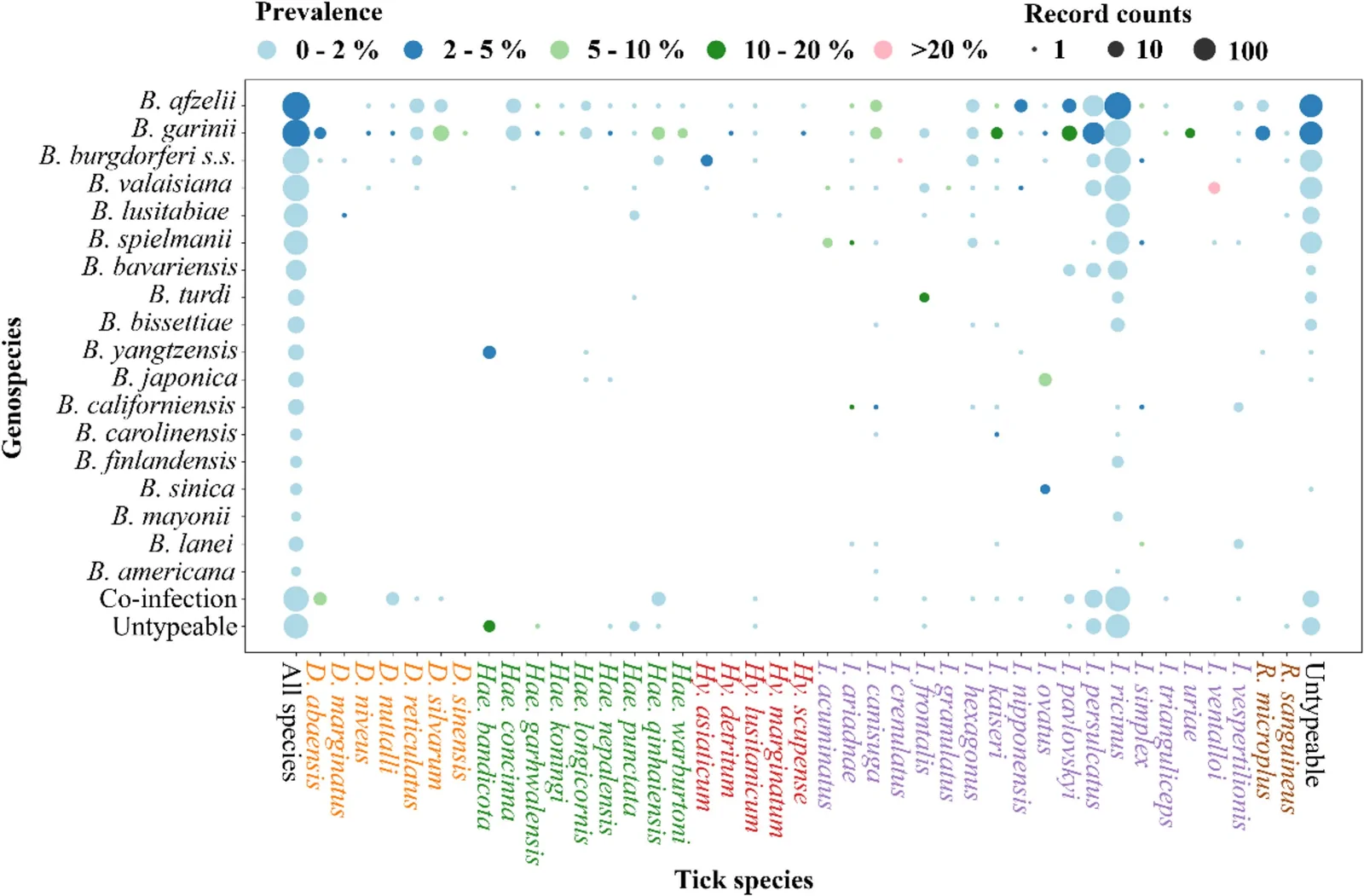
Vector-genospecies associations and prevalence patterns of *B. burgdorferi* s.l. across Eurasian tick species. Bubble size represents the number of prevalence records (sampling intensity). Bubble color indicates infection prevalence within each tick-genospecies combination, categorized into five intervals: 0–2%, 2–5%, 5–10%, 10–20%, and > 20%. Genospecies are organized along the y-axis, tick species along the x-axis. Empty cells indicate absence of detection or sampling. Co-infection refers to samples with multiple genospecies detected simultaneously; Untypeable represents *B. burgdorferi* s.l. positive samples that could not be assigned to specific genospecies.

### 3.3 Climate and host richness drive overall prevalence

At the continental scale, *B. burgdorferi* s.l. prevalence was directly influenced by climate and small mammal richness (Fig. 5). Temperature of the wettest quarter had a positive direct effect (β = 0.172, *P* < 0.001), as did small mammal richness (β = 0.139, *P* < 0.001). Avian and medium-to-large mammal richness showed no significant direct effects. Temperature also had a positive indirect effect through small mammal richness (indirect effect: 0.071). Land cover and landscape variables did not affect prevalence directly but exerted small indirect effects through forest fragmentation or host richness (indirect effect: 0.041). Standardized and unstandardized path coefficients for all structural equation models are presented in Tables S2-S4.

**Fig. 5:**
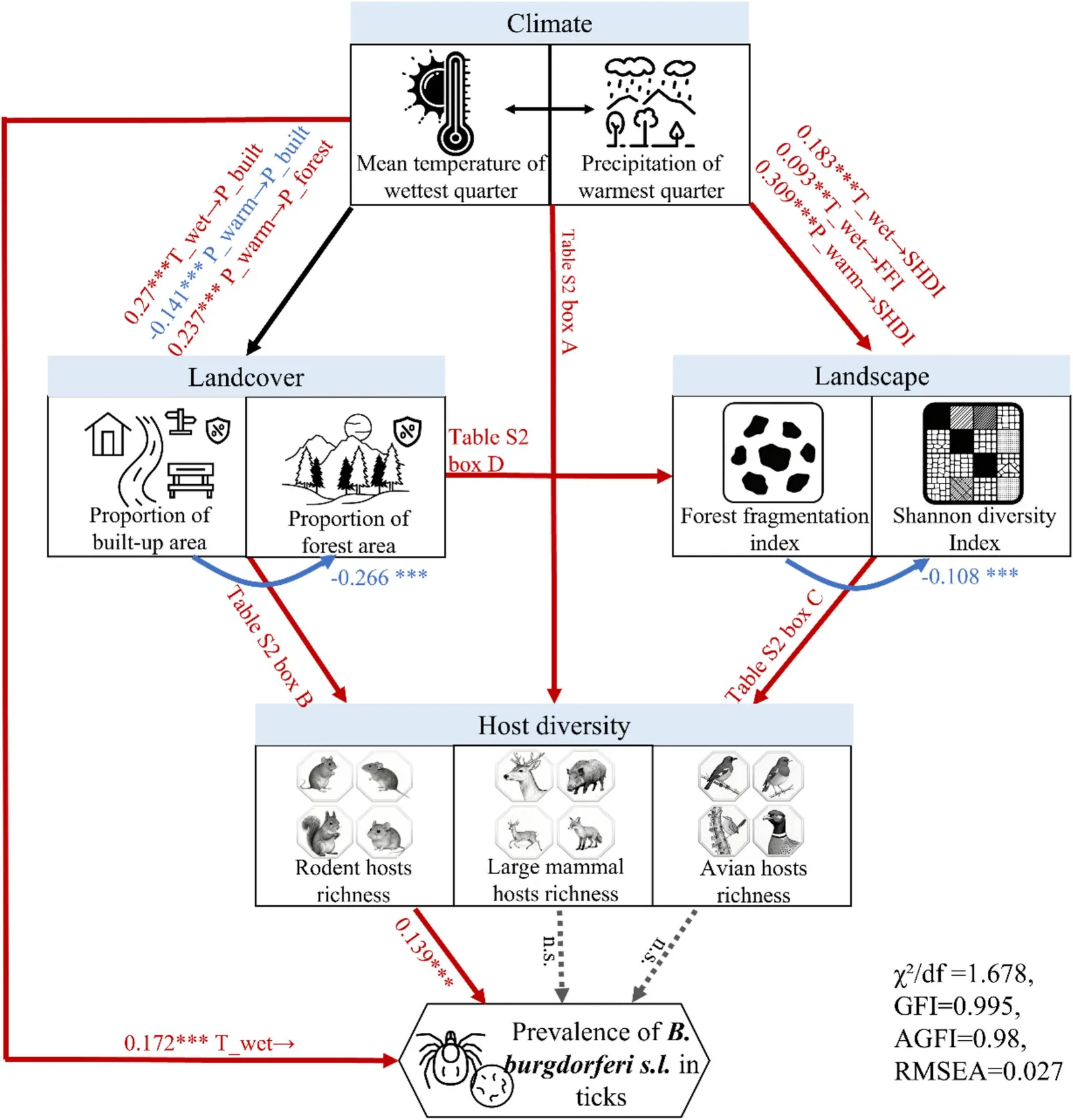
Structural equation model showing the associations of climate, land cover, landscape structure, and host diversity with the prevalence of *B. burgdorferi* s.l. Solid red arrows indicate significant positive pathways, solid blue arrows indicate significant negative pathways, and dashed black arrows indicate nonsignificant pathways. Numbers along the pathways represent standardized path coefficients. Labels adjacent to the pathways indicate where the corresponding parameter estimates are reported: Boxes A-D of Supplementary Table S2. One, two, and three asterisks denote *P* < 0.05, *P* < 0.01, and *P* < 0.001, respectively. (For interpretation of the references to color in this figure legend, the reader is referred to the web version of this article.)

### 3.4 *B. afzelii*: negatively associated with rodent richness

*B. afzelii* exhibited distinct ecology with multiple direct pathways (Fig. 6a). Small mammal richness showed a significant negative direct effect on prevalence (β = −0.197, *P* < 0.001). Climate also exerted positive direct influences. Prevalence was directly influenced by temperature of the wettest quarter (β = 0.149, *P* < 0.01) and precipitation of the warmest quarter (β = 0.132, *P* < 0.01). Landscape structure had contrasting effects. Forest fragmentation index (FFI) positively influenced prevalence (β = 0.108, *P* < 0.05), while Shannon diversity index (SHDI) exhibited negative association (β = −0.132, *P* < 0.01). Climate and land cover exerted indirect effects through dual pathways: (i) negative indirect effects via small mammal richness and landscape diversity, and (ii) positive indirect effects via forest fragmentation. Compared with the overall *B. burgdorferi* s.l. model, *B. afzelii* exhibited a reversed small mammal effect from positive to negative and receives direct regulation by landscape structure.

**Fig. 6:**
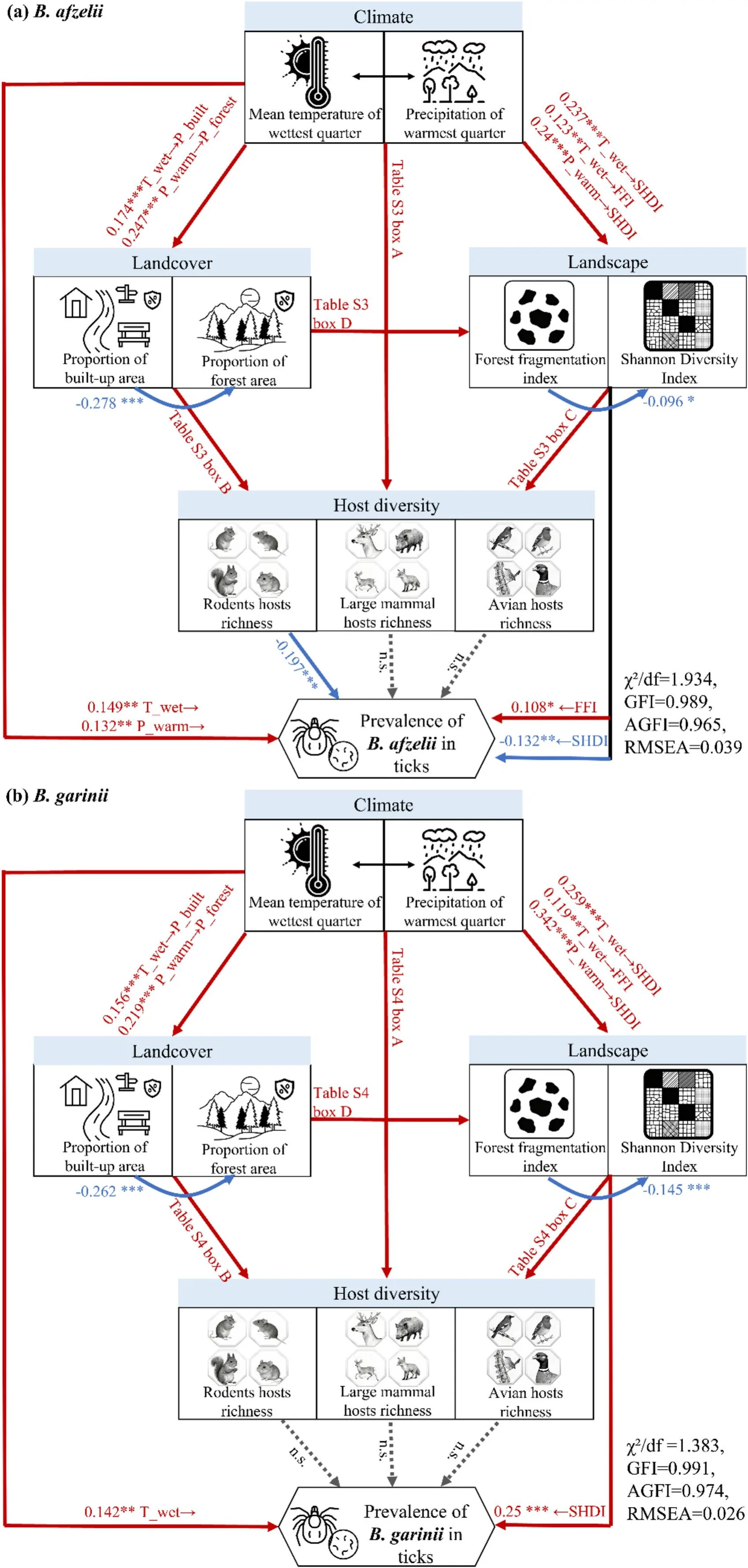
Structural equation models showing the associations of climate, land cover, landscape structure, and host diversity with the prevalence of (a) *B. afzelii* and (b) *B. garinii*. Solid red arrows indicate significant positive pathways, solid blue arrows indicate significant negative pathways, and dashed black arrows indicate nonsignificant pathways. Numbers along the pathways represent standardized path coefficients. Labels adjacent to the pathways indicate where the corresponding parameter estimates are reported: Boxes A-D of Supplementary Table S3 for panel (a) and Boxes A-D of Supplementary Table S4 for panel (b). One, two, and three asterisks denote *P* < 0.05, *P* < 0.01, and *P* < 0.001, respectively. (For interpretation of the references to color in this figure legend, the reader is referred to the web version of this article.)

### 3.5 *B. garinii*: climate-landscape control with minimal host effects

*B. garinii* transmission was related primarily to temperature and landscape diversity, with no detectable direct effects of host diversity (Fig. 6b). Prevalence increased with temperature of the wettest quarter (β = 0.142, *P* < 0.001) and with landscape diversity (β = 0.250, *P* < 0.001). Precipitation and all host richness exerted no direct effects. Land cover influenced *B. garinii* only indirectly. Higher forest area increased fragmentation (β = 0.311, *P* < 0.001) which subsequently reduced landscape diversity (β = −0.145, *P* < 0.001), which in turn affected prevalence through the SHDI. In contrast to *B. burgdorferi* s.l. and *B. afzelii*, where host community composition played a central regulatory role, *B. garinii* dynamics at the continental scale appeared to be shaped mainly by abiotic and landscape features. Host diversity acted only as an intermediate node in the climate-landscape cascade, showing no detectable direct association with prevalence.

## 4 Discussion

This study reveals that the transmission ecology of *B. burgdorferi* s.l. across Eurasia differs significantly among genospecies, particularly between *B. afzelii* and *B. garinii*. We characterized the biogeographical distribution patterns and pathogen-vector associations. *B. afzelii* is predominant in Central and Western Europe, while *B. garinii* exhibits transcontinental transmission characteristics. These distinct patterns are strongly associated with pathogen-vector-host specificity. Structural Equation Modeling (SEM) was employed to dissect genospecies-specific transmission pathways. *B. afzelii* prevalence was associated with multiple factors, including climate, host diversity, and landscape patterns. In contrast, *B. garinii* prevalence was associated primarily with climate and landscape patterns, with no detectable direct association with host diversity. This divergence suggests that effective Lyme disease monitoring and control must adopt genospecies-specific strategies, especially in the context of ongoing climate and land-use change.

### 4.1 Different *B. burgdorferi* s.l. genospecies exhibit distinct patterns of regional dominance

These geographic patterns are broadly consistent with previous European meta-analyses and recent continental-scale datasets, which identified *B. afzelii* and *B. garinii* as widely reported genospecies but documented substantial regional variation in their prevalence [19], [32], [38]. Their distributions are closely associated with the geographical ranges of competent tick vectors, with *I. ricinus* serving as the principal vector in Europe [19], [38] and *I. persulcatus* playing an important role in northern and eastern Asia [5], [32].

Rodent species richness exhibits a significant negative correlation with *B. afzelii* prevalence at the continental scale. This pattern may arise through multiple ecological pathways. At the community level, increased rodent diversity could regulate pathogen transmission through two complementary processes. Where rodent communities harbor species with heterogeneous reservoir competence, tick bites become distributed across a broader array of hosts, potentially diluting encounters with highly competent reservoirs. This diversity-mediated encounter reduction aligns with the dilution effect framework supported by North American *B. burgdorferi* s.s. systems, though its applicability to Eurasia has remained contentious [25], [26], [27]. Concurrently, interspecific competition in species-rich systems may numerically suppress dominant reservoir hosts, reducing both their population density and per-capita tick burden [26], [39]. Competitive redistribution of host abundance and diversification of tick feeding patterns may act in concert to attenuate transmission. However, the observed negative correlation may partly reflect habitat-mediated covariation rather than direct causality. Landscapes that support high rodent diversity, such as structurally complex and climatically buffered mature forests, may simultaneously alter tick activity through fine-scale microclimatic conditions, including vapor pressure deficit and humidity [9], [10], [22]. The SEM framework controls for broad-scale forest cover and landscape structure, yet fine-scale habitat attributes remain potential confounders that cannot be fully disentangled at continental spatial grains. Distinguishing among these pathways will require integrating species-level reservoir competence assessments, fine-scale microhabitat characterization, and experimental manipulations of host community composition. These approaches remain challenging across Eurasian spatial extents but are essential for mechanistic resolution. Our results suggest that the longstanding controversy over biodiversity-disease relationships in European Lyme disease systems may largely reflect insufficient taxonomic resolution in previous analyses rather than a genuine absence of diversity-mediated regulation. *B. afzelii* depends preferentially on rodent reservoir hosts, making its transmission dynamics particularly sensitive to rodent community structure [40]. When all genospecies are aggregated under the *B. burgdorferi* s.l. complex, the host-utilization patterns of other genospecies, particularly the avian-associated ecology of *B. garinii*, may obscure the rodent diversity signal specific to *B. afzelii* [14]. This genospecies-stratified perspective underscores the necessity of taxonomically resolved analyses in biodiversity-disease research.

Forest fragmentation was associated with *B. afzelii* prevalence through opposing direct and indirect pathways. The positive direct pathway may reflect edge-related changes in the abundance of competent rodent reservoirs and tick host encounter rates [9], [12]. Simultaneously, fragmentation may indirectly suppress transmission by promoting rodent community diversity and thereby activating diversity-mediated regulatory mechanisms [41]. However, our results indicate that the density-mediated amplification effect outweighs the diversity-mediated suppression effect in fragmented landscapes. It suggests that fragmentation selectively favors competitively dominant rodent species whose numerical dominance drives pathogen transmission. Landscape homogenization resulting from agricultural intensification and urbanization may further increase disease risk [13], [42]. Reduced landscape diversity not only directly elevates *B. afzelii* prevalence but also constrains small mammal diversity, thereby weakening whatever diversity-mediated suppression. These findings highlight the importance of landscape configuration, consistent with previous landscape epidemiological studies [43], [44]. They also caution against assuming that biodiversity conservation necessarily reduces disease risk, because evidence for dilution in European Lyme disease systems remains context dependent [27], [41].

*B. garinii* prevalence was positively associated with landscape diversity but shows no significant association with local host diversity. Heterogeneous landscapes support structurally diverse ecotones that favor avian communities[45]. Passerine birds can serve as reservoirs of *B. garinii* and transport infected ticks among breeding, stopover, and migratory sites [46], [47]. However, the geographic scale of our analysis is inadequate for characterizing avian-associated transmission dynamics, although it is sufficient to capture rodent-mediated patterns. This limitation arises because avian infection status integrates exposure across multiple migratory stopover sites and breeding areas. Consequently, local-scale avian richness gradients are epidemiologically uninformative for *B. garinii* prevalence patterns. Climate change is likely to further complicate *B. garinii* transmission dynamics. As anthropogenic climate change disrupts avian migration routes, alters breeding phenology, and degrades stopover habitats, *B. garinii* endemic areas may undergo more rapid and spatially complex redistribution than genospecies transmitted by small mammals with restricted home ranges.

The prevalence of all genospecies is positively associated with temperature during the wettest quarter. However, *B. afzelii* showed an additional positive association with precipitation during the warmest quarter, consistent with previously reported genospecies-specific environmental niches in Europe [19]. This precipitation is critical for *B. afzelii* transmission because it maintains microhabitat humidity necessary for tick survival while promoting rodent foraging activity. In contrast, *B. garinii* prevalence showed no significant association with precipitation. This difference may partly reflect the mobility of avian hosts, which can integrate pathogen and tick exposure across multiple locations [46], [47].

Under conditions of global environmental change, our findings support a shift from generalized surveillance toward genospecies-specific management strategies. For *B. afzelii* transmission hotspots, management could prioritize landscape heterogeneity and connectivity through habitat restoration and defragmentation initiatives [12], [48]. Maintaining diverse host communities may contribute to functional dilution, although evidence from European Lyme disease systems remains context dependent [27], [28], [41]. For *B. garinii* hotspots, surveillance could incorporate avian migration periods and ecotonal habitats, together with targeted risk communication for people undertaking outdoor activities during migratory and breeding periods [46], [47].

This study employs an updated Eurasian dataset to provide a comprehensive continental-scale assessment of *B. burgdorferi* s.l. transmission ecology at the genospecies level. The genospecies-stratified Structural Equation Modeling (SEM) framework offers a methodological refinement over traditional aggregated approaches by revealing how transmission drivers vary among genospecies with divergent host-utilization patterns. We incorporated host biodiversity partitioned by taxonomic groups aligned with genospecies-specific reservoir ecology. This taxonomically resolved approach helps clarify longstanding debates regarding biodiversity-disease relationships by demonstrating that aggregated analyses can obscure genospecies-specific ecological signals. Integrating human, animal, pathogen, and environmental information into surveillance is consistent with current One Health approaches to the management of bacterial zoonoses [49].

Several limitations warrant consideration. Heterogeneous spatial sampling effort may bias effect size estimates and limit generalizability to undersampled regions. Path estimates may be affected by important factors not measured including fine-scale anthropogenic disturbance, host behavioral traits, local vector composition, and seasonal and interannual climate variability. The cross-sectional observational design precludes assessment of temporal dynamics and does not permit causal inference regarding the identified relationships. The identified pathways should therefore be interpreted as theory-informed associations and further evaluated using longitudinal, multilevel, or spatially explicit studies.

## 5 Conclusions

In conclusion, the ecological correlates of *B. burgdorferi* s.l. prevalence vary by genospecies. *B. afzelii* prevalence was associated with climate, rodent diversity, and landscape configuration, whereas *B. garinii* prevalence was linked primarily to temperature and landscape diversity, with no detectable direct relationship with host richness at the spatial scale analyzed. These differences suggest that future Lyme disease surveillance would benefit from integrating genospecies-specific ecological information with landscape management to better coordinate biodiversity conservation, climate adaptation, and public health priorities.

## CRediT authorship contribution statement

**Yifan Sun:** Writing – original draft, Visualization, Validation, Software, Methodology, Data curation, Conceptualization. **Yinsheng Zhang:** Methodology, Data curation. **Jinchen Wang:** Data curation. **Ruying Fang:** Data curation. **Yiyang Guo:** Data curation. **Sen Li:** Supervision, Methodology, Funding acquisition, Conceptualization.

## Funding

This research was funded by the 10.13039/501100001809National Natural Science Foundation of China (No. 42477465, No. 42311530697).

## Declaration of competing interest

The authors declare that they have no known competing financial interests or personal relationships that could have appeared to influence the work reported in this paper.

## Data Availability

The dataset used in this study is publicly available in Figshare at https://doi.org/10.6084/m9.figshare.29098673.
