# Supplementary material for: Host diversity and landscape structure drive genospecies-specific Lyme disease risk across Eurasia
Source: One Health. 2026 Sep 22;23:101593. doi: 10.1016/j.onehlt.2026.101593 (PMC13634300; doi:10.1016/j.onehlt.2026.101593)
Supplement: Supplementary file 1 [file mmc1.docx]

**Host diversity and landscape structure drive genospecies-specific Lyme disease risk across Eurasia**

Yifan Sun *et al.*

*Corresponding author. Email: [senli@hust.edu.cn](mailto:senli@hust.edu.cn)

**This word file includes:**

Figures. S1 to S3

Tables S1 to S4

Figure S1.

The a priori conceptual model developed for this study based on ecological theory and published evidence. The model illustrates the hypothesized pathways linking climate, land cover, landscape structure, host-community diversity, and *B. burgdorferi* s.l. prevalence. The accompanying table summarizes the theoretical rationale and supporting references for each hypothesized pathway. Predictor variables are grouped graphically for clarity but were entered individually into the structural equation models.

| # | Links | Rationale | Ref. |
| --- | --- | --- | --- |
| 1 | Climate → Land cover | Climate is a primary driver of biome formation and land cover distribution. Temperature and precipitation patterns determine the potential vegetation types and forest distribution in a region. For example, adequate precipitation during the warmest quarter supports forest growth and maintenance, while temperature of the wettest quarter influences growing season length and forest productivity. However, the effect of climate on built-up areas is expected to be weak, as human land-use decisions are primarily driven by socioeconomic factors rather than climate constraints. | ^[1,2]^ |
| 2 | Climate → Host diversity | Climate constrains geographic distributions and abundances of host species. Temperature affects activity periods and reproductive success. Precipitation affects water and food resources. | ^[3-6]^ |
| 3 | Climate → Landscape pattern | Climate influences landscape structure over long time scales by affecting vegetation growth patterns and natural disturbance regimes. Favorable climate conditions (adequate warmth and precipitation) may reduce forest loss and fragmentation by supporting forest regeneration and reducing environmental stress. Temperature and precipitation gradients also create environmental heterogeneity, which can increase landscape diversity. Effects are relatively weak as landscape patterns are more strongly influenced by human activities. | ^[7,8]^ |
| 4 | Climate → Prevalence | Climate directly affects *Borrelia* prevalence through its influence on tick biology and host-tick interactions. Temperature influences tick developmental rates, survival, and questing behavior. Warmer temperatures accelerate tick development and extend the period of tick activity, increasing opportunities for pathogen transmission. Temperature affects the replication rate of *Borrelia* spirochetes within ticks. The relationship may be non-linear, as extremely high temperatures can reduce tick survival and activity. Precipitation affects humidity levels, which are critical for tick survival during host-seeking. Higher moisture also creates favorable microhabitat conditions in leaf litter where ticks quest for hosts. | ^[9-11]^ |
| 5 | Land cover → Host diversity | Land cover composition acts as the primary habitat filter determining which host species can persist in a landscape. Forest positively affects host diversity by providing essential habitat and food resources, particularly for forest-specialist species among rodents, medium-large mammals (e.g., deer), and birds. Built-up area generally reduces host diversity through habitat loss, but the relationship is complex. | ^[12-14]^ |
| 6 | Land cover → Landscape pattern | Land cover determines landscape metrics geometrically. Built-up area increases fragmentation | ^[15-17]^ |
| 7 | Land cover → Prevalence | Land cover directly influences *Borrelia* prevalence through multiple pathways. Forest provides the humid, shaded microhabitats essential for tick survival, particularly in the leaf litter layer where ticks quest for hosts. However, the relationship may be scale-dependent: at local scales, forest interior may have lower host-tick contact rates than forest edges. Built-up area has complex effects: moderate urbanization can increase edge habitat and create forest-human interfaces where tick-host-human contact rates are elevated, but high urbanization reduces suitable tick habitat through microclimate modification (lower humidity, higher temperature variability). | ^[12,18]^ |
| 8 | Host diversity → Prevalence | According to the dilution effect hypothesis, higher host diversity reduces pathogen prevalence by including incompetent or less competent host species without transmitting *Borrelia*. However, empirical evidence for dilution effects in Lyme disease systems is mixed. The outcome likely depends on local community composition and spatial scale. | ^[19-26]^ |
| 9 | Landscape pattern → Host diversity | Landscape pattern affects host diversity through habitat connectivity and heterogeneity mechanisms. Forest fragmentation has contrasting effects depending on host group and spatial scale. Shannon's Diversity Index represents habitat heterogeneity, which generally supports higher host diversity by providing diverse resource types and microhabitats. | ^[14]^ |
| 10 | Landscape pattern → Prevalence | Fragmentation creates edge habitats where hosts congregate, increasing tick-host contact. Fragmented landscapes have more natural-human interfaces. Edges have altered microclimates. | ^[12,14]^ |

Figure S2.

The distribution of prevalence data and administrative resolution across countries. Shading intensity on the map indicates the number of prevalence records available for each country. Pie charts overlaid on each country illustrate the distribution of data across different administrative divisions. Countries are grouped by geographical regions (Western Europe, Northern Europe, Central Europe, Southern Europe, and Eastern Europe). The bar chart at the top shows the mean prevalence across countries.

Figure S3.

Geographic (map) and statistical (raincloud plot) distribution of *B. burgdorferi* s.l. prevalence in different life and behavioral stages. (a-f) Questing larvae, questing nymph, questing adult, feeding larvae, feeding nymph, feeding adult, respectively.

Table S1.

List of variables and data sources.

| Variable Category | Variables | Data Source | Spatial Resolution | Website |
| --- | --- | --- | --- | --- |
| Climate | Temperature of the wettest quarter (℃) | WorldClim v2.1 | 5 arc-minute | https://www.worldclim.org/ |
|  | Precipitation of the warmest quarter (mm) | WorldClim v2.1 | 5 arc-minute | https://www.worldclim.org/ |
| Landcover | Proportion of forest area (%) | EarthEnv | 5 arc-minute | http://www.earthenv.org/ |
|  | Proportion of built-up area (%) | EarthEnv | 5 arc-minute | http://www.earthenv.org/ |
| Landscape | Forest fragmentation index | Global Forest Fragmentation | 5 arc-minute | https://figshare.com/s/21dbf1f50250aeb7f5a0 |
|  | Shannon diversity index | MODIS Land Cover | 5 arc-minute | https://doi.org/10.5067/MODIS/MCD12Q1.061 |
| Host diversity | Avian hosts richness | IUCN Red List | 5 arc-minute | https://www.iucnredlist.org/ |
|  | Rodent hosts richness | IUCN Red List | 5 arc-minute | https://www.iucnredlist.org/ |
|  | Large mammal species richness | IUCN Red List | 5 arc-minute | https://www.iucnredlist.org/ |
| Prevalence | Prevalence | Literature Review | Point Data | https://doi.org/10.6084/m9.figshare.29098673.v1 |

Table S2.

Standardized and unstandardized direct paths of all ecosystem drivers, including: climate (T_wet and P_warm), landcover (P_built and P_forest), landscape pattern (FFI and SHDI), and host diversity (Rich_A, Rich_R, and Rich_LM) on prevalence of *Borrelia burgdorferi* s.l. in ticks (Fig.5). This table includes all significant path, and those variables which were allow to covary. Information on variables included in box A-C in Fig. 5 is highlighted in the column "Box". *= P < 0.05, **= P < 0.01, and ***= P < 0.001. T_wet = mean temperature of wettest quarter; P_warm = precipitation of warmest quarter; P_built = proportion of built-up area; P_forest = proportion of forest area; Rich_A = avian hosts richness; Rich_R = rodent hosts richness; Rich_LM = large mammal hosts richness; FFI = forest fragmentation index; SHDI = shannon diversity index.

| Box | Predictors |  | Response | Standardized coefficients | Regression weights | P-value |
| --- | --- | --- | --- | --- | --- | --- |
|  | T_wet | → | P_built | 0.27 | 1.046 | *** |
|  | P_warm | → | P_built | -0.141 | -0.026 | *** |
|  | P_warm | → | P_forest | 0.237 | 0.07 | *** |
|  | T_wet | → | SHDI | 0.183 | 0.009 | *** |
|  | T_wet | → | FFI | 0.093 | 0.005 | ** |
|  | P_warm | → | SHDI | 0.309 | 0.001 | *** |
|  | T_wet | ←→ | P_warm | 0.313 | 203.436 | *** |
| D | P_forest | → | FFI | 0.32 | 0.003 | *** |
| D | P_built | → | FFI | 0.186 | 0.003 | *** |
|  | P_built | → | P_forest | -0.266 | -0.423 | *** |
|  | FFI | → | SHDI | -0.108 | -0.093 | *** |
| A | T_wet | → | Rich_A | 0.439 | 2.618 | *** |
| A | T_wet | → | Rich_LM | 0.426 | 0.420 | *** |
| A | T_wet | → | Rich_R | 0.455 | 0.629 | *** |
| A | P_warm | → | Rich_A | 0.094 | 0.027 | *** |
| A | P_warm | → | Rich_LM | 0.077 | 0.004 | *** |
| B | P_forest | → | Rich_A | 0.194 | 0.187 | *** |
| B | P_forest | → | Rich_R | 0.136 | 0.031 | *** |
| B | P_forest | → | Rich_LM | 0.257 | 0.041 | *** |
| C | FFI | → | Rich_A | 0.11 | 11.499 | *** |
| C | FFI | → | Rich_R | 0.307 | 7.458 | *** |
| C | FFI | → | Rich_LM | 0.168 | 2.893 | *** |
| C | SHDI | → | Rich_A | 0.208 | 25.282 | *** |
| C | SHDI | → | Rich_R | 0.09 | 2.539 | *** |
| C | SHDI | → | Rich_LM | 0.212 | 4.255 | *** |
|  | T_wet | → | Prevalence of *B. burgdorferi* s.l. | 0.172 | 0.005 | *** |
|  | Rich_R | → | Prevalence of *B. burgdorferi* s.l. | 0.139 | 0.003 | *** |

Table S3.

Standardized and unstandardized direct paths of all ecosystem drivers, including: climate (T_wet and P_warm), landcover (P_built and P_forest), landscape pattern (FFI and SHDI), and host diversity (Rich_A, Rich_R, and Rich_LM) on prevalence of *Borrelia afzelii* in ticks (Fig.5). This table includes all significant path, and those variables which were allow to covary. Information on variables included in box A-C in Fig. 5 is highlighted in the column "Box". *= P < 0.05, **= P < 0.01, and ***= P < 0.001. T_wet = mean temperature of wettest quarter; P_warm = precipitation of warmest quarter; P_built = proportion of built-up area; P_forest = proportion of forest area; Rich_A = avian hosts richness; Rich_R = rodent hosts richness; Rich_LM = large mammal hosts richness; FFI = forest fragmentation index; SHDI = shannon diversity index.

| Box | Predictors |  | Response | Standardized coefficients | Regression weights | P-value |
| --- | --- | --- | --- | --- | --- | --- |
|  | T_wet | → | P_built | 0.174 | 0.791 | *** |
|  | P_warm | → | P_forest | 0.247 | 0.09 | *** |
|  | T_wet | → | SHDI | 0.237 | 0.012 | *** |
|  | T_wet | → | FFI | 0.123 | 0.007 | ** |
|  | P_warm | → | SHDI | 0.240 | 0.001 | *** |
|  | T_wet | ←→ | P_warm | 0.151 | 74.403 | *** |
| D | P_forest | → | FFI | 0.272 | 0.002 | *** |
| D | P_built | → | FFI | 0.230 | 0.003 | *** |
|  | P_built | → | P_forest | -0.278 | -0.416 | *** |
|  | FFI | → | SHDI | -0.096 | -0.084 | * |
| A | T_wet | → | Rich_A | 0.339 | 2.106 | *** |
| A | T_wet | → | Rich_LM | 0.374 | 0.364 | *** |
| A | T_wet | → | Rich_R | 0.471 | 0.677 | *** |
| B | P_forest | → | Rich_LM | 0.105 | 0.015 | *** |
| C | FFI | → | Rich_A | 0.187 | 19.534 | *** |
| C | FFI | → | Rich_R | 0.286 | 6.891 | *** |
| C | FFI | → | Rich_LM | 0.166 | 2.700 | *** |
| C | SHDI | → | Rich_A | 0.367 | 43.577 | *** |
| C | SHDI | → | Rich_R | 0.219 | 5.996 | *** |
| C | SHDI | → | Rich_LM | 0.293 | 5.437 | *** |
|  | T_wet | → | Prevalence of *B. afzelii* | 0.149 | 0.002 | ** |
|  | P_warm | → | Prevalence of *B. afzelii* | 0.132 | 0.001 | ** |
|  | Rich_R | → | Prevalence of *B. afzelii* | -0.197 | -0.002 | *** |
|  | FFI | → | Prevalence of *B. afzelii* | 0.108 | 0.023 | * |
|  | SHDI | → | Prevalence of *B. afzelii* | -0.132 | -0.032 | ** |

Table S4.

Standardized and unstandardized direct paths of all ecosystem drivers, including: climate (T_wet and P_warm), landcover (P_built and P_forest), landscape pattern (FFI and SHDI), and host diversity (Rich_A, Rich_R, and Rich_LM) on prevalence of *Borrelia garinii* in ticks (Fig.5). This table includes all significant path, and those variables which were allow to covary. Information on variables included in box A-C in Fig. 5 is highlighted in the column "Box". *= P < 0.05, **= P < 0.01, and ***= P < 0.001. T_wet = mean temperature of wettest quarter; P_warm = precipitation of warmest quarter; P_built = proportion of built-up area; P_forest = proportion of forest area; Rich_A = avian hosts richness; Rich_R = rodent hosts richness; Rich_LM = large mammal hosts richness; FFI = forest fragmentation index; SHDI = shannon diversity index.

| Box | Predictors |  | Response | Standardized coefficients | Regression weights | P-value |
| --- | --- | --- | --- | --- | --- | --- |
|  | T_wet | → | P_built | 0.156 | 0.675 | *** |
|  | P_warm | → | P_forest | 0.219 | 0.078 | *** |
|  | T_wet | → | SHDI | 0.259 | 0.015 | *** |
|  | T_wet | → | FFI | 0.119 | 0.007 | ** |
|  | P_warm | → | SHDI | 0.342 | 0.001 | *** |
|  | T_wet | ←→ | P_warm | 0.173 | 88.931 | *** |
| D | P_forest | → | FFI | 0.311 | 0.003 | *** |
| D | P_built | → | FFI | 0.215 | 0.003 | *** |
|  | P_built | → | P_forest | -0.262 | -0.401 | *** |
|  | FFI | → | SHDI | -0.145 | -0.139 | *** |
| A | T_wet | → | Rich_A | 0.345 | 2.278 | *** |
| A | T_wet | → | Rich_LM | 0.352 | 0.360 | *** |
| A | T_wet | → | Rich_R | 0.516 | 0.759 | *** |
| A | P_warm | → | Rich_A | 0.065 | 0.023 | ** |
| B | P_forest | → | Rich_LM | 0.089 | 0.014 | *** |
| C | FFI | → | Rich_A | 0.161 | 17.674 | *** |
| C | FFI | → | Rich_R | 0.219 | 5.350 | *** |
| C | FFI | → | Rich_LM | 0.090 | 1.525 | * |
| C | SHDI | → | Rich_A | 0.392 | 44.751 | *** |
| C | SHDI | → | Rich_R | 0.209 | 5.320 | *** |
| C | SHDI | → | Rich_LM | 0.348 | 6.150 | *** |
|  | T_wet | → | Prevalence of *B. garinii* | 0.142 | 0.002 | *** |
|  | SHDI | → | Prevalence of *B. garinii* | 0.250 | 0.052 | *** |

[1]	J. Wu, J. Luo, H. Zhang, S. Qin, M. Yu, Projections of land use change and habitat quality assessment by coupling climate change and development patterns, Science of The Total Environment 847 (2022). <https://doi.org/10.1016/j.scitotenv.2022.157491>.

[2]	P.S. Roy, R.M. Ramachandran, O. Paul, P.K. Thakur, S. Ravan, M.D. Behera, C. Sarangi, V.P. Kanawade, Anthropogenic land use and land cover changes—a review on its environmental consequences and climate change, Journal of the Indian Society of Remote Sensing 50(8) (2022) 1615-1640. <https://doi.org/10.1007/s12524-022-01569-w>.

[3]	D. Thom, W. Rammer, T. Dirnböck, J. Müller, J. Kobler, K. Katzensteiner, N. Helm, R. Seidl, A. Mori, The impacts of climate change and disturbance on spatio‐temporal trajectories of biodiversity in a temperate forest landscape, Journal of Applied Ecology 54(1) (2016) 28-38. <https://doi.org/10.1111/1365-2664.12644>.

[4]	M.T.P. Coelho, E. Barreto, T.F. Rangel, J.A.F. Diniz-Filho, R.O. Wüest, W. Bach, A. Skeels, I.R. McFadden, D.W. Roberts, L. Pellissier, N.E. Zimmermann, C.H. Graham, The geography of climate and the global patterns of species diversity, Nature 622(7983) (2023) 537-544. <https://doi.org/10.1038/s41586-023-06577-5>.

[5]	C. Howard, P.A. Stephens, J.W. Pearce‐Higgins, R.D. Gregory, S.G. Willis, The drivers of avian abundance: Patterns in the relative importance of climate and land use, Global Ecology and Biogeography 24(11) (2015) 1249-1260. <https://doi.org/10.1111/geb.12377>.

[6]	T. Montràs-Janer, A.J. Suggitt, R. Fox, M. Jönsson, B. Martay, D.B. Roy, K.J. Walker, A.G. Auffret, Anthropogenic climate and land-use change drive short- and long-term biodiversity shifts across taxa, Nature Ecology & Evolution 8(4) (2024) 739-751. <https://doi.org/10.1038/s41559-024-02326-7>.

[7]	C. Dobbs, C. Nitschke, D. Kendal, Assessing the drivers shaping global patterns of urban vegetation landscape structure, Science of The Total Environment 592 (2017) 171-177. <https://doi.org/10.1016/j.scitotenv.2017.03.058>.

[8]	K.M. Bigsby, M.R. McHale, G.R. Hess, Urban morphology drives the homogenization of tree cover in baltimore, md, and raleigh, nc, Ecosystems 17(2) (2013) 212-227. <https://doi.org/10.1007/s10021-013-9718-4>.

[9]	P.A. Nuttall, Climate change impacts on ticks and tick-borne infections, Biologia 77(6) (2021) 1503-1512. <https://doi.org/10.1007/s11756-021-00927-2>.

[10]	L. Gilbert, The impacts of climate change on ticks and tick-borne disease risk, Annu Rev Entomol 66 (2021) 373-388. <https://doi.org/10.1146/annurev-ento-052720-094533>.

[11]	J.C. Burtis, P. Sullivan, T. Levi, K. Oggenfuss, T.J. Fahey, R.S. Ostfeld, The impact of temperature and precipitation on blacklegged tick activity and Lyme disease incidence in endemic and emerging regions, Parasites & Vectors 9(1) (2016). <https://doi.org/10.1186/s13071-016-1894-6>.

[12]	G. Perez, S. Bastian, A. Agoulon, A. Bouju, A. Durand, F. Faille, I. Lebert, Y. Rantier, O. Plantard, A. Butet, Effect of landscape features on the relationship between *Ixodes ricinus* ticks and their small mammal hosts, Parasites & Vectors 9(1) (2016). <https://doi.org/10.1186/s13071-016-1296-9>.

[13]	M.A. Diuk-Wasser, M.C. VanAcker, M.P. Fernandez, W. Reisen, Impact of land use changes and habitat fragmentation on the eco-epidemiology of tick-borne diseases, Journal of Medical Entomology 58(4) (2021) 1546-1564. <https://doi.org/10.1093/jme/tjaa209>.

[14]	F. Guo, T.C. Bonebrake, L. Gibson, Land-use change alters host and vector communities and may elevate disease risk, EcoHealth 16(4) (2018) 647-658. <https://doi.org/10.1007/s10393-018-1336-3>.

[15]	M. Masoudi, D.R. Richards, P.Y. Tan, Assessment of the influence of spatial scale and type of land cover on urban landscape pattern analysis using landscape metrics, Journal of Geovisualization and Spatial Analysis 8(1) (2024). <https://doi.org/10.1007/s41651-024-00170-8>.

[16]	H. Dadashpoor, P. Azizi, M. Moghadasi, Land use change, urbanization, and change in landscape pattern in a metropolitan area, Science of The Total Environment 655 (2019) 707-719. <https://doi.org/10.1016/j.scitotenv.2018.11.267>.

[17]	H. Li, J. Peng, L. Yanxu, H. Yi’na, Urbanization impact on landscape patterns in beijing city, China: A spatial heterogeneity perspective, Ecological Indicators 82 (2017) 50-60. <https://doi.org/10.1016/j.ecolind.2017.06.032>.

[18]	R. Rosà, V. Andreo, V. Tagliapietra, I. Baráková, D. Arnoldi, H. Hauffe, M. Manica, F. Rosso, L. Blaňarová, M. Bona, M. Derdáková, Z. Hamšíková, M. Kazimírová, J. Kraljik, E. Kocianová, L. Mahríková, L. Minichová, L. Mošanský, M. Slovák, M. Stanko, E. Špitalská, E. Ducheyne, M. Neteler, Z. Hubálek, I. Rudolf, K. Venclikova, C. Silaghi, E. Overzier, R. Farkas, G. Földvári, S. Hornok, N. Takács, A. Rizzoli, Effect of climate and land use on the spatio-temporal variability of tick-borne bacteria in Europe, International Journal of Environmental Research and Public Health 15(4) (2018). <https://doi.org/10.3390/ijerph15040732>.

[19]	R.S. Ostfeld, F. Keesing, Biodiversity and disease risk: The case of Lyme disease, Conservation Biology 14(3) (2001) 722-728. <https://doi.org/10.1046/j.1523-1739.2000.99014.x>.

[20]	K. LoGiudice, R.S. Ostfeld, K.A. Schmidt, F. Keesing, The ecology of infectious disease: Effects of host diversity and community composition on Lyme disease risk, Proceedings of the National Academy of Sciences USA (100) (2003) 567-571.

[21]	S. Chen, S.E. McFarlane, Reappraisal of the dilution and amplification effect framework: A case study in Lyme disease, Ecology and Evolution 15(8) (2025). <https://doi.org/10.1002/ece3.71969>.

[22]	S.C. Ruyts, D. Landuyt, E. Ampoorter, D. Heylen, S. Ehrmann, E.C. Coipan, E. Matthysen, H. Sprong, K. Verheyen, Low probability of a dilution effect for Lyme borreliosis in Belgian forests, Ticks and Tick-borne Diseases 9(5) (2018) 1143-1152. <https://doi.org/10.1016/j.ttbdis.2018.04.016>.

[23]	V. Ratti, J.M. Winter, D.I. Wallace, Dilution and amplification effects in Lyme disease: Modeling the effects of reservoir-incompetent hosts on *Borrelia burgdorferi* sensu stricto transmission, Ticks and Tick-borne Diseases 12(4) (2021). <https://doi.org/10.1016/j.ttbdis.2021.101724>.

[24]	S.J. Halsey, J.R. Miller, Maintenance of *Borrelia burgdorferi* among vertebrate hosts: A test of dilution effect mechanisms, Ecosphere 11(2) (2020). <https://doi.org/10.1002/ecs2.3048>.

[25]	S.C. Ruyts, E.V.Y. Ampoorter, E.C. Coipan, L. Baeten, D. Heylen, H. Sprong, E. Matthysen, K. Verheyen, Diversifying forest communities may change Lyme disease risk: Extra dimension to the dilution effect in Europe, Parasitology 143(10) (2016) 1310-1319. <https://doi.org/10.1017/s0031182016000688>.

[26]	P.H. Kelly, J. Davis, Y. Tan, H.M. Marick, A. Davidson, A. Estrada-Peña, J.C. Moïsi, J.H. Stark, Occurrence and prevalence of host-seeking *Ixodes ricinus* nymphs infected with *Borrelia burgdorferi* (sensu lato) genospecies in Europe (1999–2022), Current Research in Parasitology & Vector-Borne Diseases 8 (2025). <https://doi.org/10.1016/j.crpvbd.2025.100297>.
